# Anti-Mullerian-hormone levels during pregnancy and postpartum

**DOI:** 10.1186/1477-7827-11-60

**Published:** 2013-07-11

**Authors:** Angela Köninger, Alexis Kauth, Boerge Schmidt, Markus Schmidt, Guelen Yerlikaya, Sabine Kasimir-Bauer, Rainer Kimmig, Cahit Birdir

**Affiliations:** 1 Department of Gynecology and Obstetrics, University of Duisburg-Essen, Hufelandstrasse 55, 45122 Essen, Germany; 2 Institute for Medical Informatics, Biometry and Epidemiology (IMIBE), University of Duisburg-Essen, Hufelandstrasse 55, 45122 Essen, Germany; 3 Department of Gynecology and Obstetrics, Klinikum Duisburg GmbH, Zu den Rehwiesen 9-11, 47055 Duisburg, Germany; 4 Department of Gynecology and Obstetrics, University of Vienna, Spitalgasse 23, 1090 Wien, Austria

**Keywords:** Anti-mullerian hormone, Pregnancy, Postpartum, Ovarian suppression, Ovarian reserve

## Abstract

**Background:**

The number of unintentionally childless couples is increasing as more couples seek to conceive for the first time in the third or fourth decade of the woman’s life. Determination of ovarian reserve is an essential component of infertility assessment. The Anti-Müllerian-Hormone (AMH) seems to be the most reliable predictor of ovarian reserve. In this study we analyzed AMH in a cohort of pregnant women without fertility impairment to determine age-dependent decline and possible AMH fluctuations during pregnancy and postpartum.

**Methods:**

A total of 554 healthy women aged 16 to 47 years without history of infertility or previous surgery on the ovaries were enrolled in the study between 1995 and 2012. In 450 women, a single measurement of AMH was taken during pregnancy, allowing for cross sectional analysis of trimester- and age-related differences in AMH levels. For another 15 women longitudinal data on AMH levels for all trimesters was recorded. In addition, for 69 women AMH was measured at the time just before and after delivery, and for another 20 AMH was measured just before delivery and once on each of the first four days after delivery. We used AMH-Gen-II ELISA (Beckman Coulter, Immunotech, Webster, USA) for the assessment of AMH levels. Non-parametric statistical tests were used to compare AMH levels between age groups, trimesters and postpartum.

**Results:**

Comparison between the trimesters revealed a significant difference in AMH values at each trimester (first trimester: 1.69 ng/ml (IQR 0.71–3.10), second trimester: 0.8 ng/ml (IQR 0.48–1.41), third trimester: 0.5 ng/ml (IQR 0.18–1.00)). AMH significantly dropped during the course of pregnancy and immediately after delivery, whereas an increase was observed over the first four days postpartum. Women, greater than or equal to 35 years, showed significant lower AMH levels than those <35 years across all trimesters.

**Conclusions:**

AMH levels decrease during pregnancy. The decline in AMH levels during pregnancy indicates ovarian suppression. AMH levels recover quickly after delivery. AMH levels assessed in pregnant women are not an accurate indicator of ovarian reserve, since AMH levels during pregnancy seem not to be independent of gestational age.

## Background

There has been an increase in the number of unintentionally childless couples with the increase in the average age of first conception. One explanation for this might be a significantly reduced ovarian reserve in older women. It is well known that the ovarian reserve steadily decreases from the onset of puberty until menopause [1]. Iatrogenic reduction of ovarian reserve may be caused by surgeries to remove endometriosis and cysts; these procedures are more frequently performed in today’s era of minimally invasive surgery [2]. In addition, genetic disorders may also play a role in early loss of ovarian reserve [3].

There are various known parameters for assessing ovarian reserve, including ovarian volume, antral follicle count, follicle stimulating hormone (FSH) and Inhibin B at the beginning of the menstrual cycle, as well as Anti-Müllerian-Hormone (AMH) [4,5]. AMH is produced by the granulosa cells of small antral and prae-antral follicles, and the quantity of AMH corresponds to the size of the pool of these follicles in animal models as well as in the human ovary [6-8]. Furthermore, AMH inhibits the initiation of follicle growth and the FSH-dependent selection process [9-11]. AMH correlates very well with the sonographically measurable antral follicle count and has been shown to decrease with increasing age [12]. AMH is currently considered to be the most reliable predictor of the ovarian reserve [7,12,13]. By contrast to established factors like age, FSH, estradiol and Inhibin B, AMH reflected the pool of primary and secondary follicles and indicates the age-dependent decline of the follicle pool before other factors change [13]. The number of oocytes retrieved in IVF protocols strongly correlated with the ovarian reserve. AMH predicted more precisely the quantity of oocyte retrieval than other factors [14]. AMH levels seemed not to vary during the menstrual cycle [15-17]. No changes in AMH levels over the course of pregnancy had heretofore been reported [18]. In addition, the intake of hormonal substances had up to now been believed to have no significant effect on AMH levels [19].

Most recent studies investigating AMH levels before pregnancy were conducted in populations of women receiving in-vitro fertilization. Gnoth et al. [14] demonstrated that the level of AMH before ovarian stimulation correlated with the number of punctured oocytes, which led to the acceptance of low AMH levels as a predictor of poor ovarian response. Clinical pregnancy rates, however, showed no correlation with AMH levels. Our clinical experience is consistent with this finding, showing that some women with very low or even undetectable AMH levels get pregnant naturally and give birth at full term.

Since recent research has argued that AMH levels decline only with respect to age and not because of hormonal changes (e.g. contraception), we sought to examine AMH levels to determine the age-dependent decline in a cohort of pregnant women without fertility impairment. The further aim was to evaluate possible AMH fluctuations during the course of pregnancy and postpartum. We therefore sought to investigate the reliability of AMH as a marker of ovarian reserve in a highly hypogonadotropic state such as pregnancy.

## Methods

### Study population

554 healthy women aged 16 to 47 years (mean±SD: 30.8 ± 6.2 years) without history of infertility or previous surgery on the ovaries were enrolled in the study. All patients were treated in the clinic for obstetrics and gynaecology at the University Hospital of Essen, Germany, between 1995 and 2012. All of them conceived naturally without artificial infertility treatment. None of the patients had a history of chemotherapy or radiation treatment. Women with a history of ovarian surgery were excluded from the study.

Patients were enrolled in the study in order as they appeared in the clinic. Informed written consent was obtained from all women, and the study was approved by the local research ethics committee (number 11–4643). The Helsinki Declaration was followed throughout the study.

### Study design

#### AMH levels during pregnancy

AMH was studied prospectively in the third trimester (≥ 29th week of gestation) in 339 women presenting in our clinic between 2011 and 2012. Since AMH levels were very low, we retrospectively studied serum samples taken during the first (n=58; ≤ 14th week of gestation) and second (n=53; 15–28 weeks of gestation) trimester from women seen in our clinic between 2007 and 2011. AMH levels were analyzed separately for each trimester according to age groups (≤27 years, 28–34 years and ≥35 years). Sample size was adequate to detect even small differences in AMH levels between trimesters as previously suggested [18].

In addition, to determine the relevance of AMH levels longitudinally during the course of pregnancy, 15 patients with blood samples from each trimester, presenting in our clinic between 1995 and 2001, were evaluated for AMH levels retrospectively.

### AMH levels peripartal

69 women were studied prospectively between 2011 and 2012 by taking one blood sample during admission for delivery and one additional blood sample within the first four days after delivery. Another 20 patients had blood samples taken at the time just before delivery and additionally during each of the first four days after delivery to assess AMH levels during this period.

### Sampling of serum

Blood samples (9 ml) were collected from each woman using S-Monovettes (Sarstedt AG & Co.), stored at 4°C and processed within 4 hours to avoid blood cell lysis. Blood fractionation was carried out by centrifugation for 10 minutes at 2500xg. Subsequently, 3 to 4 ml of the upper phase, constituting blood serum, were removed for the assessment of AMH levels.

### Determination of AMH levels

For AMH determination, we used serum samples in which the enzymatically amplified two-site AMH-Gen-II ELISA was applied (Beckman Coulter, Immunotech, Webster, Texas, USA). Briefly, undiluted serum samples and controls were dispensed into the wells coated with anti-AMH antibody, followed by the addition the anti-AMH detection antibody labelled with biotin. After washing, 100 μl of the streptavidin-horseradish peroxidase (HRP) was added, followed by the addition of 100 μl substrate solution containing TMB for 8–12 minutes. The degree of enzymatic turnover of the substrate was determined by dual wavelength absorbance measurement at 450 nm and between 600 and 630 nm using an automatic ELISA reader (Bio-Rad, Hercules, CA). The absorbance measured was directly proportional to the concentration of AMH in the samples which was calculated from the calibration curve. The results were expressed in ng/ml according to the established standard curve. Concentrations below 0.08 ng/ml were considered undetectable.

### Statistical analysis

As AHM levels were found not to be normally distributed in the study population, results are reported as median and interquartile ranges (IQR). The Kruskal-Wallis test with *post hoc* Wilcoxon rank-sum test was used to analyze differences in AMH levels between trimesters and age groups. Differences between pre- and postpartum AMH levels were analyzed using the Wilcoxon signed rank test. For the analysis of differences between AMH levels assessed before delivery and at the first four days postpartum, as well as for analyzing trends prospectively during trimesters, Friedman rang sum test with *post hoc* Wilcoxon-Nemenyi-McDonald-Thompson test was used.

The level of statistical significance was set at α = 0.05. For *post hoc* multiple testing we adjusted the alpha-level using Bonferroni’s method (α_BF_~0.017 for comparing trimesters and α_BF_~0.005 for comparing AHM levels before delivery and the first four days postpartum).

All analyses were performed using the R statistical package version 2.15.2 and SAS 9.2 (SAS Institute, Cary NC) [20].

## Results

### AMH levels during pregnancy stratified by trimesters

**Figure 1:**
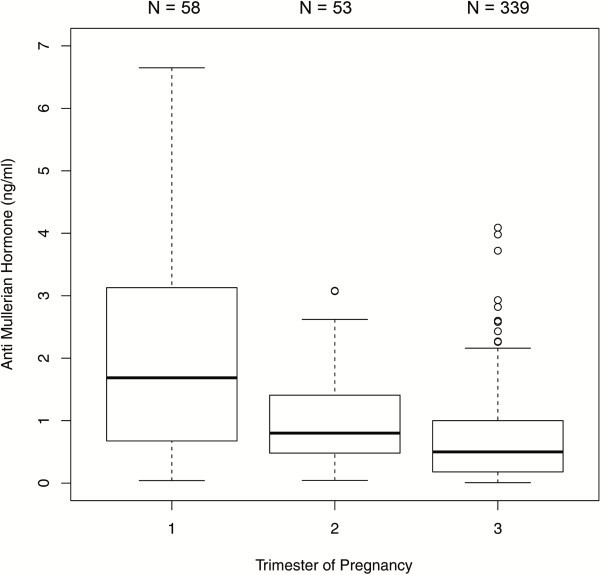
Boxplots illustrating the distribution of AMH levels for each trimester including all age groups.

**Figure 2:**
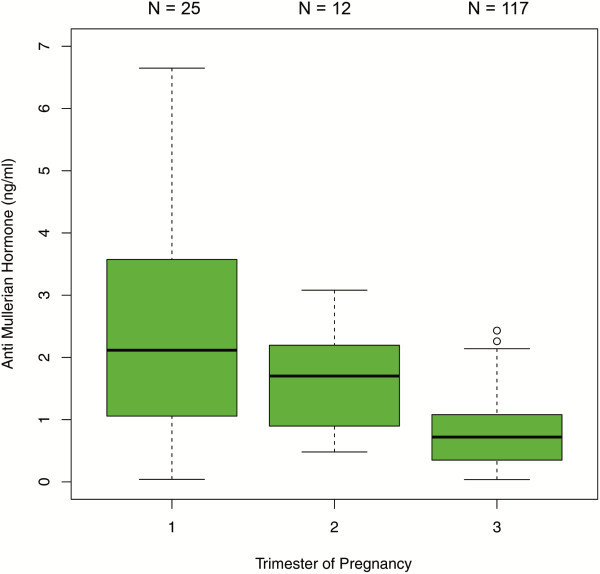
**Boxplots illustrating the distribution of AMH levels for each trimester including women aged** ≤**27 years.**

**Figure 3:**
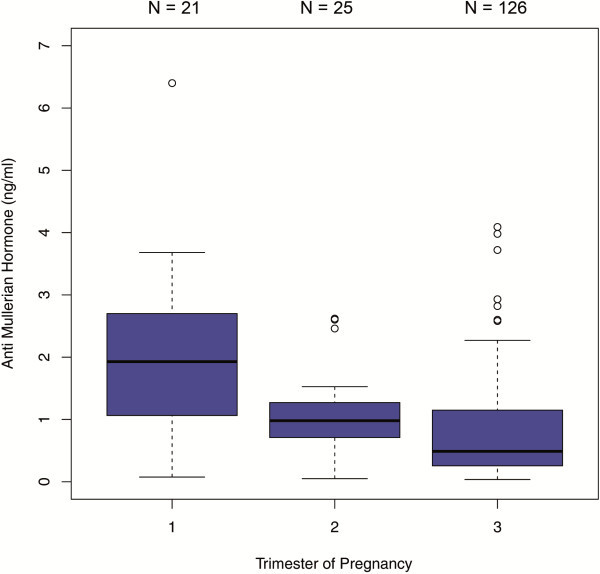
**Boxplots illustrating the distribution of AMH levels for each trimester including women aged 28**–**34 years.**

**Figure 4:**
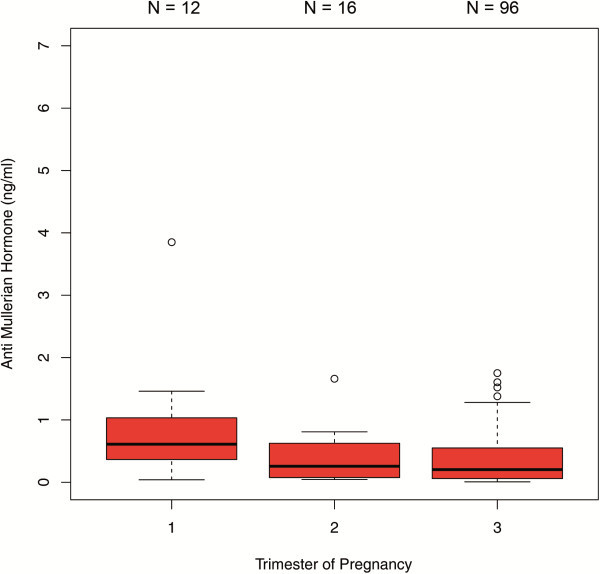
**Boxplots illustrating the distribution of AMH levels for each trimester including women aged** ≥ **35 years.**

The comparisons between the trimesters in the 450 patients with single AMH measurements revealed significant differences in AMH median values between each trimester. AMH dropped significantly from the first to the second trimester [1.69 ng/ml (IQR 0.71 - 3.10) versus 0.80 ng/ml (IQR 0.48 - 1.41); p < 0.001] and from the second to the third trimester [0.80 ng/ml (IQR 0.48- 1.41) versus 0.50 ng/ml (IQR 0.18 - 1.00); p < 0.01] (Figure 1). When this sample was analyzed stratified by age groups, the following results were obtained: In the age group of ≤27 years (group 1), there was a significant difference between the median values obtained for the first and third trimester as well as for the second and third trimester (p < 0.001) (Figure 2). In the 28–34 year age group (group 2), differences in AMH levels turned out to be significant between each trimester, with decreasing median values from first to second trimester (p < 0.01) and from second to third trimester (p=0.016) (Figure 3). In the ≥35 years age group (group 3) there were no significant differences in AMH median values observed between all trimesters (p > 0.05) (Figure 4). An overview of AMH median values and the respective IQR for patients with single AMH measurements stratified by age group and trimester is given in table 1.

**Table 1:** **AMH median values and interquartile ranges** (**IQR**) **stratified by age group and trimester** (**n**=**450**)

**Age**	**First trimester**	**Second trimester**	**Third trimester**
*≤27 years*	2.12 ng/ml (IQR 1.06–3.57)	1.70 ng/ml (IQR 1.00–2.18)	0.72 ng/ml (IQR 0.35–1.08)
*28–34 years*	1.93 ng/ml (IQR 1.06–2.70)	0.98 ng/ml (IQR 0.71–1.27)	0.49 ng/ml (IQR 0.26–1.14)
*≥ 35 years*	0.61 ng/ml (IQR 0.37–1.01)	0.26 ng/ml (IQR 0.08–0.62)	0.20 ng/ml (IQR 0.07–0.55)

### AMH levels assessed longitudinally during pregnancy

**Figure 5:**
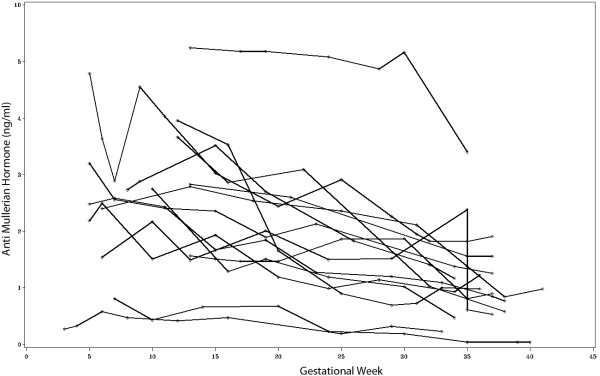
**Trends of AMH levels during the course of pregnancy in women with multiple AMH measurements (n=15).**

In 15 patients AMH levels were measured in multiple blood samples during the entire course of pregnancy. Figure 5 shows that the overall trend of AMH levels during the course of pregnancy is inversely related to gestational age. Using the first measured value per patient in each trimester for analysis, statistically significant differences were observed between the first and the third [2.73 ng/ml (IQR 1.87 - 3.25) versus 1.38 ng/ml (IQR 1.03 - 1.72); p < 0.0001] as well as between the second and the third trimester [2.36 ng/ml (IQR 1.59 - 2.95) versus 1.38 ng/ml (IQR 1.03 - 1.72); p < 0.01].

### AMH levels during pregnancy stratified by age

In the 450 patients with single AMH measurements, analyses stratified by age revealed that the AMH levels in age groups 1 and 2 did not differ significantly across all trimesters. In contrast, age group 3 showed a significantly lower AMH median value compared to group 1 (p < 0.01 in the first trimester; p < 0.001 in the second trimester; p < 0.0001 in the third trimester) as well as compared to group 2 (p < 0.01 in the first trimester; p < 0.001 in the second trimester; p < 0.0001 in the third trimester) (Table 1).

### AMH levels pre- and postpartal

**Figure 6:**
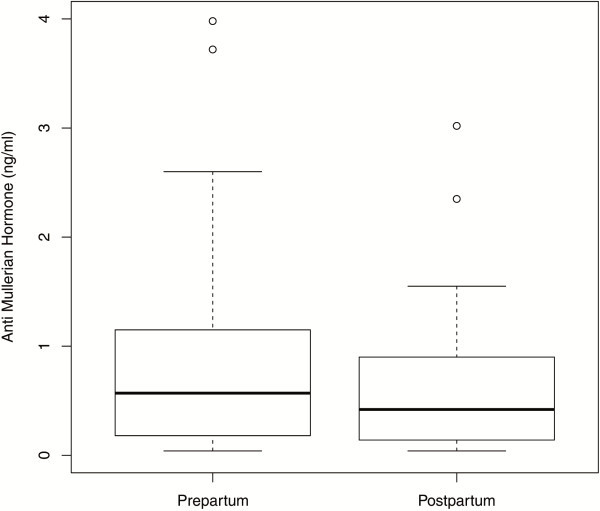
**Boxplots illustrating the distribution of AMH levels in women with AMH measurements prepartum and postpartum (n**=**69).**

In the 69 patients, for whom the AMH levels were determined immediately before delivery and during the first four days postpartum, there was a significant difference between pre- and postpartal median values, showing that AMH levels continue decreasing shortly after delivery [0.57 ng/ml (IQR 0.18–1.15) versus 0.42 ng/ml (IQR 0.14–0.90); (p < 0.0001)] (Figure 6).

**Figure 7:**
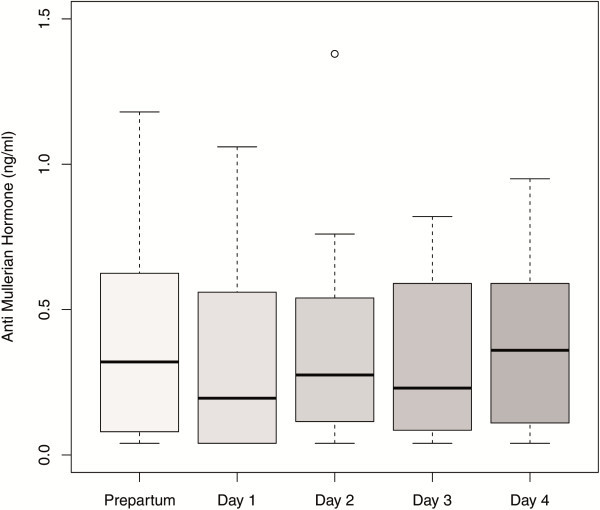
**Boxplots illustrating the distribution of AMH levels in women with AMH measurements prepartum and at each of the first four days postpartum (n=20).**

In the 20 patients for whom AMH levels were determined before delivery and at each of the first four days postpartum there was a statistically significant increase of AMH median values only between day one and day four [0.20 ng/ml (IQR 0.04–0.56) versus 0.36 ng/ml (IQR 0.15–0.58); p<0.005] (Figure 7).

## Discussion

Although AMH has been considered to be a surrogate marker of ovarian reserve, up to now only a few studies have examined the role of AMH in pregnancy and postpartum. Here we studied AMH in a population of 554 pregnant women without fertility impairment to explore age-dependent AMH levels and changes in AMH during the course of pregnancy and postpartum. AMH levels during pregnancy seem to be inversely related to age. The main finding of our study is that AMH levels decrease with increasing gestational age in a cross sectional study population as well as longitudinally. Furthermore, AMH levels seem to drop postpartum compared to levels measured shortly before delivery, but there is some evidence that AMH levels increase again during the first four days postpartal.

### Unexpectedly low AMH

We first examined AMH levels in women who gave birth after a full term pregnancy. We observed unexpectedly low AMH levels in the study sample. In women younger than 28 years, the median value determined in the third trimester was 0.72 ng/ml and the median value of all age groups was 0.5 ng/ml.

Kelsey et al.[1] described an age-related standard curve of AMH over a lifetime based on a variety of studies. The authors found a peak at 25.4 years at an average of 4 ng/ml. According to studies by Gnoth et al. [14], the threshold between normal and poor response of ovarian stimulation in a cohort receiving in-vitro- fertilization was 1.26 ng/ml. In comparison with the infertile cohort of Gnoth et al. [14] and the standard curve determined by Kelsey et al. [1], the median value of 0.50 ng/ml in the third trimester observed in our study seemed to be unexpectedly low, suggesting that AMH levels during pregnancy do not represent a reliable predictor of ovarian reserve.

### AMH decline

Since AMH levels found at the end of pregnancy were unexpectedly low, we compared AMH levels in different trimesters. In this context, we demonstrated that AMH levels decreased significantly with increasing gestational age. We confirmed these results longitudinally in a cohort, measuring AMH levels at each trimester in women for which blood sampling was performed every second week during pregnancy. A comparable study with different results was published by La Marca et al. [18] who determined AMH in every trimester of pregnancy as well as in a small cohort of non-pregnant women in a cross sectional analysis (control group: n=15, group with AMH measurements in the first trimester: n=27, group examined in the second trimester: n=21, group with AMH measurements in the third trimester: n=13, postpartal group: n=8). La Marca et al. saw a trend towards lower AMH levels in the third trimester without statistical significance [18].

The diverging results observed by La Marca et al. [18] may reflect their relatively small study population (n=76, i.e. controls and pregnant women) that was not well powered to detect small differences in AMH levels between trimesters. By contrast, we examined 450 pregnant women in cross-sectional analysis. In addition to the cross-sectional examination, we were able to confirm our results longitudinally in a cohort of 15 patients with measurements in each of the trimesters. To our knowledge, this is the first study showing an AMH decline during pregnancy including longitudinal data.

### Age dependence

Analyzing AMH levels according to age showed that AMH levels in all trimesters did not differ significantly in the two age groups below 35 years, whereas women aged ≥ 35 years had significantly lower AMH levels. In contrast, the course of AMH drop was different within every age group. In women aged ≤ 34 years, a significant decrease of AMH between different trimesters was observed. In older women aged ≥35 years only non-significant differences between the different trimesters were obtained which might be due to the overall low ovarian reserve of the older patients. With regard to the cross sectional analyses across all age groups, a decrease in AMH median values of approximately 50% could be observed in subsequent trimesters.

### Pre- and postpartum

In a subset of women, we were able to compare AMH levels pre- and postpartal, finding a significant drop of AMH after delivery. Furthermore, a more detailed analysis of AMH levels within the first four days postpartal suggests that a further decrease of AMH one day postpartum is followed by a significant increase up to day four postpartum.

La Marca et al. [18] also explored postpartal AMH levels, but in a cross sectional design. The authors did not observe differences between postpartal, i.e. two or three days after delivery, and AMH levels in pregnancy. The differing study results of La Marca et al. [18] might be explained by the lack of detailed longitudinal data as used in the study presented here. Furthermore, we observed a significant increase in AMH levels on day 4 postpartum, whereas in the study of La Marca blood samples were taken only on days 2 and 3 postpartum.

As our study suggests, the ovary seems to be suppressed in pregnancy and relegated to a nearly prepubertal quiescent state. In prepubertal state, there are low AMH values despite a high ovarian reserve [21]. AMH production recovered after delivery, showing a small but significant increase by the fourth day postpartum, suggesting an immediate recovery of follicular development after delivery.

Our data are consistent with a study by Weghofer et al. [22] who investigated AMH levels in a cohort of 210 women without evidence of impaired fertility 14 days after delivery in order to determine an age-related difference. Across three different age groups (18–30 years, 30–36 years and 37–40 years; 70 patients each), AMH levels fourteen days after delivery were much lower than among infertile women. The authors concluded that the hormonal suppression of pregnancy led to a reduction in AMH levels which did not correlate with the size of the pool of follicles, but with ovarian function.

Weghofer et al. [22] examined the women only once after delivery, whereas in our study we also examined pregnant women. Going beyond Weghofer et al. [22] we were able to describe an AMH increase within a few days after delivery.

Our study results offer biological plausibility. Folliculogenesis in pregnancy seems to be inhibited, so that even the most primordial follicles seem to be in a resting state and AMH is no longer produced. The loss of the hormone producing placenta could be the cause for another drop in AMH levels after delivery and before a subsequent rise. Thus, in addition to the hormones progesterone and estrogen, other placental factors may amplify the strong ovarian suppression and could explain the further drop of AMH after delivery. One explanation might be that FSH is strongly suppressed in pregnancy as well as in the first three days after delivery [18]. We looked for a possible candidate which could be responsible for this FSH suppression peripartal. Beyond the sexual steroids, the activin-binding protein Follistatin suppresses FSH synthesis and secretion. As shown by Rae et al. [23] Follistatin increases during pregnancy and is still enhanced during the first day after delivery, returning to values similar to those in late pregnancy at the second day after birth. Because of the short half-time of Follistatin, the authors concluded that the placenta may not be the only source of Follistatin. Other authors assume a role in tissue repair by Follistatin to explain the short-term postpartal increase [24]. In the context of our study results, Follistatin may contribute to further AMH decrease shortly after delivery. Further research is needed to examine a potential interaction of Follistatin or other possible candidates for FSH suppression, Anti-Mullerian- Hormone and ovarian suppression during pregnancy.

From our perspective, ovarian suppression may function as fertility preserving. Our results give support to the hypothesis of ovarial protection in full term pregnancy, using AMH as a serum marker. This is in accordance with the observation that women reaching full-term pregnancies get postmenopausal significantly later in their lifetime compared to nulliparous women. This has been reported in a variety of epidemiological studies from different ethnicities [25-32].

### Limitations

Despite several strengths, our study is limited by the fact that we were not able to measure AMH levels during pregnancy and peripartal in all patients to obtain longitudinal data at different stages in pregnancy and beyond for the whole study population. Therefore, our results have to be confirmed in a prospective population-based pregnancy cohort with detailed information on AMH levels during pregnancy as well as during the first days postpartum.

## Conclusions

AMH levels decline during pregnancy. Our study shows that AMH cannot be accepted as a parameter of ovarian reserve that remains unchanged irrespective of the hormonal circumstances of women, e.g. pregnancy. Furthermore, a full-term pregnancy may lead to suppression of the ovary as shown by a significant decrease in AMH levels during pregnancy and the quick increase postpartum. This may lead to misinterpretation of the actual ovarian reserve in pregnancy and postpartum.

## Abbreviations

AMH: Anti-Mullerian hormone; FSH: Follicle stimulating hormone.

## Competing interests

The authors declare that they have no competing interests.

## Authors’ contributions

A.Kö. gave substantial contributions to conception and design, to acquisition of data and interpretation of data, drafting the article and final approval of the version to be published. A.Ka. gave substantial contributions to acquisition of data, revising the article critically for important intellectual content and final approval of the version to be published. B.S. gave substantial contributions to analysis and interpretation of data, revising the article critically for important intellectual content and final approval of the version to be published. G.Y. and M.S. gave substantial contributions to acquisition of data, revising the article critically for important intellectual content and final approval of the version to be published. S. KB. gave substantial contributions to conception and design, drafting the article, revising it critically for important intellectual content and final approval of the version to be published. R.K. gave substantial contributions to acquisition of data, revising the article critically for important intellectual content and final approval of the version to be published. C.B. gave substantial contributions to acquisition of data, revising the article critically for important intellectual content and final approval of the version to be published. All authors read and approved the final manuscript.
